# Prevalence of migraine and co-morbid psychiatric disorders among students of Cumhuriyet University

**DOI:** 10.1186/1129-2377-14-34

**Published:** 2013-04-11

**Authors:** Murat Semiz, İlteriş Ahmet Şentürk, Hatice Balaban, Ayşegül Kartal Yağız, Önder Kavakçı

**Affiliations:** 1 Department of Psychiatry, Sivas State Hospital, Sivas, TR-58140, Turkey; 2 Department of Neurology, Cumhuriyet University Faculty of Medicine, Sivas, Turkey; 3 Department of Psychiatry, Cumhuriyet University Faculty of Medicine, Sivas, Turkey

**Keywords:** Migraine, Prevalence, Psychiatric co-morbidity, University students, Epidemiology

## Abstract

**Backround:**

The aim of this study was to investigate the prevalence of migraine and associated psychiatric disorders among university students at Cumhuriyet University of Sivas in Turkey.

**Methods:**

A total of 1601 university students participated in this study and answered the questionnaires. The study was conducted in three stages: the self-questionnaire, the neurological evaluation, and the psychiatric evaluation. In the first stage, the subjects completed a questionnaire to assess migraine symptoms. In the second stage, the subjects who reported having migraines underwent a detailed neurological evaluation conducted by a neurologist to confirm the diagnosis. In the final stage, the subjects with migraines completed a psychiatric examination using the structured clinical interview for DSM IV-R Axis I.

**Results:**

The self-reported migraine prevalence rate was 13.7%, and the actual prevalence rate of migraine among the university students was calculated to be 10.6% (n = 169). When the results obtained with the SCID-I were examined, a current SCID-I psychiatric diagnosis was found in 39 (23.1%) of the 169 subjects with migraines. A total of 73 (43.2%) students with migraines had a lifetime SCID-I psychiatric diagnosis.

**Conclusions:**

The results of this study indicate that migraines were highly prevalent among university students in Turkey with comorbid psychiatric disorders. Treatment strategies must be developed to manage these comorbidities.

## Background

Migraine is an important health problem due to the frequency and accompanying morbidity, which includes disability and the loss of performance [1-3]. Migraine has a lifetime prevalence of 12–18%, which has been shown to be both age- and gender-dependent in community-based studies worldwide [4]. The one year prevalence of migraine was reported to be between 12.4% and 12.6% in nationwide studies [5,6]

Studies have shown consistently that migraineurs report a lower quality of life than do those without migraines and that these reductions extend to physical health, mental health, social functioning and academic performance [7,8]. Migraine-type headaches are also prevalent among university students and have a profound impact on school performance in university students [8]. This impact is more evident among migrainous students than students with episodic tension-type headaches (ETTH), with a 62,7% decrease in capacity versus 24.4%. Moreover, students with migraine-type headaches missed more school than students with ETTH [9]. These results reveal the importance of migraine headaches in university students. Despite these findings, there are few studies focused on the prevalence of migraine in university students in Turkey [2,10].

An expanding body of literature has suggested that migraine headaches are associated with higher rates of psychiatric disorders. Studies have shown significant associations between migraine and a variety of psychiatric disorders, including major depressive disorder, anxiety disorders and alcohol or drug abuse and dependence [11]. Patients with migraines, anxiety, and chronic depression also had a poor health-related quality of life. In addition, migraine, specific phobias, and panic disorder were important and independent comorbidities predicting a poor health-related quality of life [12,13]. Although migraine is prevalent among university students, previous studies have not adequately assessed comorbid psychiatric conditions among university students [14,15]. Because previous studies utilised the population admitted to the clinic when searching for the psychiatric comorbidities of migraine patients, our knowledge is restricted to migraine patients whom were not on therapy. We selected our study population by field screening to add more patients who were not on therapy for psychiatric comorbidities.

Researchers have reported the prevalence of migraines using self-reporting instruments without performing neurological evaluations [2,4,5,9]. Therefore, reliable studies in which individuals diagnosed with migraines are subjected to a detailed evaluation by a neurologist and psychiatrist are needed. Understanding the nature of the association between migraines and psychiatric disorders and other conditions has implications for diagnosis and treatment. Knowledge of the migraine risks factors and pathophysiologic mechanisms is limited, and the occurrence of comorbidities may also provide clues regarding the aetiology of migraine [16]. The aim of this study was to investigate the actual prevalence of migraine and the comorbidity of psychiatric disorders among students at Cumhuriyet University of Sivas in Turkey.

## Methods

### Participants

Cumhuriyet University (CU) was founded in 1974 in Sivas, which is located in central Anatolia in Turkey. The city’s central population was 315 000 according to the census data of 2011. Sivas, having more traditional attitudes, is located in a less-industrialised part of Turkey with low education level and high unemployment rate. CU has nine faculties and three institutes. The total number of students on the Cumhuriyet University campus was 18 904. Of these students, 8332 were female, and 10 572 were male. The subjects for this research were selected from students enrolled in a university program on the Cumhuriyet University Central Campus. The Directorate of the Cumhuriyet University Student Services selected the students according to faculty, university program, and class. Our study population was composed of 18 904 students. Of these 18 904 students, 1650 were selected using randomised stratified sampling (p = 0.005, α = 0.01 and d = 0.05).

A simple random sampling method was used to obtain a representative sample of the university population. From the 1650 students who were informed about the study, 32 students (1.9%) declined to participate in the study. Thus, 1618 students participated in the study. Of the study participants, 54.3% (878) were male, and 45.7% (740) were female, and their ages ranged from 18–27 years (mean age, 21.3 ± 2.2 years).

### Procedure

The study incorporated three stages. In the first stage, the students were asked to complete a questionnaire to establish a migraine diagnosis during a school visit. The first part of the instrument consisted of questions regarding demographic characteristics, including age, medical history, family history, family structure, family socioeconomic status, smoking habits, and alcohol use. The second part was composed of questions related to the 2004 diagnostic criteria for the International Headache Society (IHS) for migraines [17]. The ID Migraine was also used.

The subjects who reported having migraines in the screening questionnaire participated in the second stage of the study. During this stage, a neurologist conducted a full neurological evaluation to confirm the migraine diagnosis. The neurological evaluation detailed a headache history and a neurological examination. The Turkish version of the Migraine Disability Assessment Scale (MIDAS) questionnaire was then administered to the students to assess failure due to migraine. In the third stage, students with migraines completed a psychiatric examination with the Structured Clinical Interview for DSM-IV-R Axis I (SCID-I). The psychiatric examination was performed by two psychiatrists (MS, AKY).

Approval by the institutional ethical committee of the Cumhuriyet University Faculty of Medicine was obtained prior to the study and informed consent was obtained from the students.

### Measures

#### Socio-demographic data form

Questions were related to the participants’ age, gender, marital status, family income, tobacco and alcohol use.

#### Identification of migraine (ID Migraine™)

As a widely used screening instrument for identifying migraine at primary health services, the ID Migraine is a three-question screening tool for migraines that has demonstrated good validity [18]. Each of the three items relates to a central diagnostic symptom of migraine: nausea, photophobia, and interference with activities. Each question is scored dichotomously with endorsements of two or more items suggesting probable migraine sensitivity and specificity. A positive predictive value on this test has been defined as 81, 75, and 93%, respectively. The Turkish version of the ID Migraine™ screening test has previously been validated [19].

#### Migraine disability assessment scale (MIDAS)

The MIDAS questionnaire is used to gather information on disability in terms of missed days of paid work (or school), housework (chores), and non-work time. Questions are asked regarding either days of missed activity or days during which productivity was reduced by at least 50%. If productivity decreased to 50% or less, the day is considered missed [20]. The 4-point grading system for the MIDAS questionnaire is as follows: Grade I (scores ranging from 0 to 5), little or no disability; Grade II (scores ranging from 6 to 10), mild disability; Grade III (scores ranging from 11 to 20), moderate disability; and Grade IV (scores of 21 or greater), severe disability. The Turkish version of the MIDAS questionnaire was developed by Ertas et al. [21].

#### The visual analogue scale (VAS)

The VAS is a simple and commonly used method for evaluating variations in pain intensity [22]. The subjects are instructed to indicate the intensity of their pain by marking a 100-mm line anchored with terms that describe the extremes of pain intensity.

#### The structured clinical interview for DSM-IV-R (SCID-I)

According to the DSM-IV, the SCID-I is a clinical interview comprising six structured modules that are utilised by an interviewer to determine whether an individual has one or more Axis-I disorders. The average application period is 25–60 min, and the evaluation is conducted with the patient individually. During the application, the interviewer uses an administration booklet with interview questions and a scoring sheet to record the ratings. The psychiatric diagnosis is determined based on “current” and “lifetime” experiences [23]. Developed by First et al. in 1997 into a Turkish reliability study, an adaptation of the SCID-I was conducted by Özkürkçügil et al. [24]. For all diagnoses, the interviewer agreement was 98.1%, and the kappa coefficient was 0.86. For all diagnostic categories, the kappa coefficients ranged between 0.52 and 1.00 and were significant (p < 0.001).

### Statistical analyses

The statistical analyses were performed using the Statistical Package for the Social Sciences (SPSS) Version 14.00. The data for categorical variables were presented as counts and percentages; the data for continuous variables were presented as the mean and SD. A comparison of variables between the groups was performed using the Independent *t* test for numeric variables and the chi-square (*x*^2^) test for categorical data. In all analyses, p values less than 0.05 were considered significant.

## Results

A total of 1618 students participated in the study. Table 1 displays the demographic and social characteristics of these students. Migraine-type headaches were detected in 221 of 1618 (13.7%) subjects using a self-reporting instrument. Of these 221 eligible subjects, 204 accepted further evaluation. Seventeen students declined to participate in the second part of the study. However, only 169 (10.6%) of the 1601 subjects were diagnosed with migraine based on a personal neurological interview. Table 1 displays the demographic and social characteristics of the 169 students. Therefore, 1432 (89.4%) of the 1601 subjects were migraine-free. The self-reported migraine prevalence was 13.7%, whereas the migraine prevalence among the medically evaluated students was 10.6%. Of these 169 subjects with migraines, 123 (72.8%) were female, and 46 (27.2%) were male. The mean age of disease onset was 17.11 years, and the average number of attacks per month was 6.3 (min 1–max 15). In addition, 130 (76.9%) of 186 students had migraines without aura, whereas the remaining 39 students (23.1%) had migraines with aura. No significant differences were found between the socioeconomic status of those students with and without migraines (p > 0.05). Similarly, the smoking habits and alcohol use did not differ significantly between the groups. Table 2 shows the clinical characteristics of the migraines.

**Table 1:** Demographic characteristics of students

	**Total**	**Migraine**	**Non-migraine**
Age	21.30 ± 2.22	20.43 ± 1.94	21.38 ± 2.19
Sex female	878(54.3%)	123 (72.8%)	634(44.3%)
male	740(45.7%)	46 (27.2%)	798(55.7%)
Marital status			
single	1569 (97%)	165 (97.6%)	1388 (96.9%)
married	49 (3%)	4 (2.4%)	44 (3.1%)
Family history of headache			
yes	597 (36.9%)	114 (67.5%)	443 (30.9%)
no	1021 (63.1%)	55 (32.5%)	989 (69.1%)
Family history of neurological disease			
yes	24 (1.5%)	3 (1.7%)	21 (1.5%)
no	1594 (98.5%)	166 (98.3%)	1411 (98.5%)
Smoking yes	179 (11.1%)	12 (7.1%)	167 (11.6%)
no	1439 (88.9%)	157 (92.9%)	1265 (88.4%)
Alchol abuse yes	113 (7.0%)	8 (4.7%)	105 (7.2%)
no	1505 (93.0%)	161 (95.3%)	1344 (92.8%)

**Table 2:** Clinical characteristics of migraine in students

**Characteristics**	**Number/Total**
Pain level	
Mild	54/169
Moderate	88/169
Severe	27/169
Frequency of pain at three months	
0-5	87/169
6-10	74/169
11	8/169
Mean duration of attacks	
4-5 hours	68/169
6-11 hours	34/169
12-23 hours	23/169
24 hours or more	44/169
Types of migrane	
With aura	39 (23.1%)
Without aura	160 (77.9%)

The MIDAS scores showed that 59 (34.9%) students had minimal disability, with a mean score of 4. Thirty-two (18.9%) students had mild disability, with a mean score of eight. In addition, 38 (22.5%) had moderate disability, with a mean score of 14, and 40 (23.7%) had severe disability, with a mean score of 26 (Table 3).

**Table 3:** MIDAS grades of the students

**MIDAS grade**	**Number**
Grade I	59 (34.9%)
Grade II	32 (18.9%)
Grade III	38 (22.5%)
Grade IV	40 (23.7%)

When the results obtained using the SCID-I were examined, a current SCID-I psychiatric diagnosis was found in 39 (23.1%) of the 169 subjects with migraine. Two (5.1%) of these thirty-nine patients had two psychiatric diagnoses (panic disorder and depressive disorder). A total of 73 (43.2%) students with migraine had a lifetime SCID-I psychiatric diagnosis. Nine patients (12.3%) had two psychiatric diagnoses throughout their life. The current and lifetime DSM-IV Axis-I disorders are shown in Table 4.

**Table 4:** Axis I diagnoses of migraine

**Axis I comorbidities**	**Current n(%)**	**Lifetime n(%)**
Depressive disorder	17 (10,1)	36 (18,9)
Dysthymic disorder	4 (2,4)	7 (4,1)
Post-traumatic stres disorder	6 (3,5)	11 (6,5)
Panic disorder	6 (3,5)	10 (5,9)
Generalized anxiety disorder	2 (1,3)	5(2,9)
Social phobia	2 (1,3)	4 (2,4)
Obsessive compulsive disorder	3 (1,8)	7(4,1)
Bipolar disorder	2 (1,3)	2(1,3)

Migraine patients with present and lifelong psychiatric diagnoses had significantly more severe headaches (p < 0.01), higher MIDAS points (p < 0.01) and more frequent migraine attacks (p < 0.05) but showed no differences based on the migraine initiation age and mean attack time (p > 0.05) compared with the migraine patients without any psychiatric diagnosis.

## Discussion

The present study was conducted among students registered to different faculties of Cumhuriyet University, Sivas, Turkey. To date, there have been university surveys evaluating the prevalence of migraines based on self-reports, but this study appears to be the first reporting the migraine prevalence in university students with a clinical interview for migraine and co-morbid psychiatric diagnoses. Structured diagnostic interviews increase the reliability and power of the prevalence studies. The subjects with migraines were interviewed and evaluated by a neurologist and a psychiatrist in this study. The diagnosis of migraine was determined using the 2004 IHS criteria, which are utilised in epidemiological studies due to their high sensitivity [17].

The prevalence of migraines was found to be 10.6%. Migraine without aura was the most common type of migraine found in this study. The observed migraine prevalence was similar to those reported in larger population studies that used structured diagnostic interviews. There are few studies focusing on migraines among university students in Turkey showing differences in prevalence.

The migraine prevalence ranged from 7.2-21.9% in the university students of Turkey [1,2,9,25] but ranged from 6.4-48.5% in other international studies [26-29]; in some studies, higher frequencies were reported. We think that the differences might be caused by the methodological differences; different self-reporting questionnaires may reflect different findings. We think the use of a self-administered questionnaire might cause the misunderstanding of some questions, which risks subjectivity in the answers. The examination of the subjects by a neurologist for headache confirmed the screening results and excluded the other headache types. We found the migraine prevalence was 13.7% by the questionnaire evaluation but was 10.6% by the clinical evaluation used for confirmation.

Migraine is often comorbid with psychiatric disorders, such as mood disorders and anxiety disorders. Muftuoglu et al. found that migraine patients were considerably more depressed and anxious than the healthy controls [29]. We observed at least one psychiatric diagnosis in 23.1% of the migraine patients at the present time and in 43.2% of migraine patients lifelong. A previous study observed depression in 10-40% of the migraine patients and mood disorders in 13.6% of the patients [30]. In population studies, migraine sufferers are between 2.2 and 4.0 times more likely to suffer from a major depressive disorder than non-migraineurs [11]. Innamorati et al. reported that many patients with chronic migraine reported symptoms of depression and stagnation and that investigating the presence of the depressive disorder may be useful for understanding the psychology of chronic migraineurs [13]. We observed depression as the most frequent psychiatric disorder in the migraine patients, similar to the findings in the literature of 10.1%. The relationship between migraines and bipolar disorder has been studied in clinical studies [31]. Merinkangas et al. observed a 2-fold increase in bipolar disorder in migraine patients compared with non-migraineurs with bipolar disorder observed in 1.2% of their migraine patients [32]. A previous study observed the bipolar disorder prevalence to be 0.43% in Sivas province [33]. Two patients were diagnosed with bipolar disorder (1.3%) in this study.

Anxiety disorders are also associated with migraines [34]. Compared with individuals without migraines, migraineurs are at 4–5 times greater risk for generalised anxiety disorder (GAD), five times greater risk for obsessive compulsive disorder (OCD), and 3–10 times more likely to suffer from panic disorder [35,36]. We observed anxiety disorder in 11.2% of the migraine patients, with panic disorder and posttraumatic stress disorder being the most frequent, in agreement with the literature. There is increasing support in the literature for a strong association between migraine and PTSD in migraine patients [37]. High PTSD prevalence rates of 22–50% have been reported in individuals with migraines in previous studies of adult subjects fulfilling the criteria for PTSD [38,39]. The results of the present study showed that PTSD with migraine was as high in university students as in the general population.

Guidetti et al. reported that psychiatric comorbidities negatively affected the migraine patients in their 8-year, longitudinal observational study [40]. Another study reported a worse quality of life in the migraine patients with anxiety disorder and depression compared with the control group and found that the psychiatric comorbidities had negative effects on the pain severity and number of episodes [41]. Most migraine sufferers do not treat themselves in anticipation of the headache, and they often returned unused quantities of drugs to physicians. The correct assessment of anxiety, depression, and stress appear critical for developing an adequate preventive treatment strategy [42].

We found no relationship among the migraine initiation age and the psychiatric comorbidities, which might be affected by the self-reporting of the initiation age by the patients. Thirty-two people in the first phase and seventeen in the second phase (total of 49 [2.9%]) refused to join the study or were excluded because of the lack of communication. The second limitation of this study is the lack of assessment of the non-migraine group for psychiatric diagnoses. The screening for migraines with only a self-reporting scale and enrolled students from different faculties may have influenced the study results. Another limitation of our study was the inability to show the causality of the relationship between psychiatric disorders and headache-related disability because of the cross-sectional structure. Prospective studies are needed to explain this relationship in detail.

## Conclusions

In conclusion, in this study, the prevalence rate of migraines among university students at Cumhuriyet University was 10.6%. Psychiatric disorders are common in university students with migraines and correlate with the pain-related disability. The finding that the majority of the students with migraines had not visited a neurologist or a psychiatrist shows that there is a lack of diagnosis and treatment in this population. The identification and treatment of psychiatric disorders in university students with migraines are an important and represent a potentially modifiable health state. Taken together, the results of previous studies with adults and the present study suggest that the treatment of psychiatric conditions positively influence the levels of pain and migraine-related disability in university students. Therefore, most migraine patients need to be followed due to the frequent presence of psychiatric conditions. However, clinicians should share their knowledge with other specialists, and psychiatric treatment must be viewed in a multidisciplinary context to improve both the quality of life and outcome of migraine patients.

## Competing interests

The authors have no financial obligations to disclose related to this study.

## Authors’ contributions

MS and HB conceived and supervised the project and drafted the manuscript. MS, İAŞ, and AKY were responsible for data collection. OK performed the statistical analyses. All authors read and approved the final manuscript.

## References

[B1] Kurt S Kaplan Y Epidemiological and clinical characteristics of headache in university students Clin Neurol Neurosurg 2008 14 46 50 10.1016/j.clineuro.2007.09.001 17949895

[B2] Demirkırkan MK Ellidokus H Boluk E Prevalence and clinical characteristics of migraine in university students in Turkey Thoku J Exp Med 2006 14 87 92 10.1620/tjem.208.87 16340178

[B3] Adoukonou T Houinato D Kankouan J Makoutode M Paraiso M Tehindrazanarivelo A Viader F Preux PM Migraine among university students in Cotonou (Benin) Headache 2009 14 887 893 10.1111/j.1526-4610.2009.01408.x 19389138

[B4] Breslau N Rasmussen BK The impact of migraine: epidemiology, risk factors and comorbidities Neurology 2001 14 4 12 10.1212/WNL.56.1.4 11294954

[B5] Zarifoğlu M Karli N Tas¸ kapilioğlu O Can ID migraine be used as a screening test for adolescent migraine? Cephalalgia 2008 14 65 71 17986273 10.1111/j.1468-2982.2007.01470.x

[B6] Ertas M Baykan B Orhan EK Zarifoglu M Karli N Saip S Onal AE Siva A One-year prevalence and the impact of migraine and tension-type headache in Turkey: a nationwide home-based study in adults J Headache Pain 2012 14 147 157 10.1007/s10194-011-0414-5 22246025 PMC3274583

[B7] Smitherman TA McDermott MJ Buchanan EM Negative impact of episodic migraine on a university population: quality of life, functional impairment, and comorbid psychiatric symptoms Headache 2011 14 581 589 10.1111/j.1526-4610.2011.01857.x 21457242

[B8] Souza-e-Silva HR Rocha-Filho PA Headaches and academic performance in university students: a cross-sectional study Headache 2011 14 1493 1502 10.1111/j.1526-4610.2011.02012.x 22082420

[B9] Bigal ME Bigal JM Betti M Bordini CA Speciali JG Evaluation of the impact of migraine and episodic tension-type headache on the quality of life and performance of a university student population Headache 2001 14 710 719 10.1046/j.1526-4610.2001.041007710.x 11554960

[B10] Bicakci S Bozdemir N Over F Saatci E Sarica Y Prevalence of migraine diagnosis using ID Migraine among university students in southern Turkey J Headache Pain 2008 14 159 163 10.1007/s10194-008-0031-0 18427728 PMC3476199

[B11] Baskin SM Smitherman TA Migraine and psychiatric disorders: comorbidities, mechanisms, and clinical applications Neurol Sci 2009 14 61 65 10.1007/s10072-009-0071-5 19415428

[B12] Pompili M Serafini G Di Cosimo D Dominici G Innamorati M Lester D Forte A Girardi N De Filippis S Tatarelli R Martelletti P Psychiatric comorbidity and suicide risk in patients with chronic migraine Neuropsychiatr Dis Treat 2010 14 81 91 20396640 10.2147/ndt.s8467 PMC2854084

[B13] Innamorati M Pompili M Fiorillo M Lala N Negro A Del Bono SD Lester D Girardi P Martelletti P Overattachment and perceived disability in chronic migraineurs 2012 Epub ahead of print 10.1016/j.clineuro 23107164

[B14] Ferri-de-Barros JE Alencar MJ Berchielli LF Castelhano LC Jr Headache among medical and psychology students Arq Neuropsiquiatr 2011 14 502 508 10.1590/S0004-282X2011000400018 21755130

[B15] Amayo EO Jowi JO Njeru EK Headache associated disability in medical students at the Kenyatta National Hospital Nairobi East Afr Med J 2002 14 519 523 10.4314/eamj.v79i10.8813 12635756

[B16] Serafini G Pompili M Innamorati M Gentile G Borro M Lamis DA Lala N Negro A Simmaco M Girardi P Martelletti P Gene variants with suicidal risk in a sample of subjects with chronic migraine and affective temperamental dysregulation Eur Rev Med Pharmacol Sci 2012 14 1389 1398 23104655

[B17] Headache Classification Committee of the International Headache Society The international classification of headache disorders, 2nd edition Cephalalgia 2004 14 1 159

[B18] Lipton RB Dodick D Sadovsky R Kolodner K Endicott J Hettiarachchi J Harrison W ID Migraine validation study. A self-administered screener for migraine in primary care: the ID migraine validation study Neurology 2003 14 375 382 10.1212/01.WNL.0000078940.53438.83 12913201

[B19] Siva A Zarifoglu M Ertas M Saip S Karli HN Baykan B Keskinaslan A Senocak M Validity of the ID-migraine screener in the workplace Neurology 2008 14 1337 1345 10.1212/01.wnl.0000309221.85545.0d 18413587

[B20] Bigal ME Rapoport AM Lipton RB Tepper SJ Sheftell FD Assessment of migraine disability using the migraine disability assessment (MIDAS) questionnaire: a comparison of chronic migraine with episodic migraine Headache 2003 14 336 342 10.1046/j.1526-4610.2003.03068.x 12656704

[B21] Ertas¸ M, Siva A, Dalkara T, Uzuner N, Dora B, Inan L, Idiman F, Sarica Y, Selçuki D, Sirin H, Oğuzhanoğlu A, Irkec¸ C, Ozmenoğlu M, Ozbenli T, Oztürk M, Saip S, Neyal M, Zarifoğlu M, Turkish MIDAS group Validity and reliability of the Turkish migraine disability assessment (MIDAS) questionnaire Headache 2004 14 786 793 10.1111/j.1526-4610.2004.04146.x 15330825

[B22] Chapman CR Casey KL Dubner R Foley KM Gracely RH Reading AE Pain measurement: an overview Pain 1985 14 1 31 4011282 10.1016/0304-3959(85)90145-9

[B23] First MB Spitzer RL Gibbon M Williams JBW Structured clinical interview for DSM-IV Axis I disorders, clinician version (SCID-CV) 1996 Washington, DC: American Psychiatric Press Inc

[B24] Ozkürkçügil A Aydemir O Yıldız M Danacı AE Köroğlu E DSM-IV eksen I bozuklukları için yapılandırılmış klinik görüşmenin Tükçeye uyarlanması ve güvenilirlik çalışması İlac¸ ve Tedavi Dergisi 1999 14 233 236

[B25] Oztora S Korkmaz O Dagdeviren N Celik Y Caylan A Top MS Asil T Migraine headaches among university students using ID Migraine test as a screening tool BMC Neurol 2011 14 101 103 10.1186/1471-2377-11-103 PMC3176162 21854556

[B26] Galinović I Vuković V Troselj M Antić S Demarin V Migraine and tensiontype headache in medical students: a questionnaire study Coll Antropol 2009 14 169 173 19408621

[B27] Zwart JA Dyb G Holmen TL Stovner LJ Sand T The prevalence of migraine and tension-type headaches among adolescents in Norway. The Nord-Trøndelag Health Study (Head-HUNT-Youth), a large population-based epidemiological study Cephalalgia 2004 14 373 379 10.1111/j.1468-2982.2004.00680.x 15096226

[B28] Ojini FI Okubadejo NU Danesi MA Prevalence and clinical characteristics of headache in medical students of the University of Lagos, Nigeria Cephalalgia 2009 14 472 477 10.1111/j.1468-2982.2008.01766.x 19170698

[B29] Muftuoglu MN Herken H Demirci H Virit O Neyal A Alexithymic features in migraine patients Eur Arch Psychiatry Clin Neurosci 2004 14 182 186 15205972 10.1007/s00406-004-0466-5

[B30] Baskin SM Smitherman TA Comorbidity between migraine and depression: update on traditional and alternative treatments Neurol Sci 2011 14 9 13 10.1007/s10072-011-0549-9 21533704

[B31] Low NC Du Fort GG Cervantes P Prevalence, clinical correlates, and treatment of migraine in bipolar disorder Headache 2003 14 940 949 10.1046/j.1526-4610.2003.03184.x 14511270

[B32] Merikangas KR Angst J Isler H Migraine and psychopathology. Results of the Zurich cohort study of young adults Arch Gen Psychiatry 1990 14 849 853 10.1001/archpsyc.1990.01810210057008 2393343

[B33] Özdemir Ö Sivas il merkezinde iki uçlu duygudurum bozukluğunun yaygınlığı, eş tanılar ve hastaların yaşam kalitesinin incelenmesi 2005 Sivas: Yayınlanmamış Uzmanlık Tezi

[B34] Hamelsky SW Lipton RB Psychiatric comorbidity of migraine Headache 2006 14 1327 1333 10.1111/j.1526-4610.2006.00576.x 17040330

[B35] Evans RW Rosen N Migraine, psychiatric comorbidities and treatment Headache 2008 14 952 958 10.1111/j.1526-4610.2008.01074.x 18572433

[B36] Merikangas KR Merikangas JR Angst J Headache syndromes and psychiatric disorders: association and familial transmission J Psychiatr Res 1993 14 197 210 10.1016/0022-3956(93)90008-P 8366469

[B37] Balaban H Semiz M Sentürk IA Kavakçı O Cınar Z Dikici A Topaktaş S Migraine prevalence, alexithymia, and post-traumatic stress disorder among medical students in Turkey J Headache Pain 2012 14 459 467 10.1007/s10194-012-0452-7 22535148 PMC3464464

[B38] de Leeuw R Schmidt JE Carlson CR Traumatic stressors and post-traumatic stress disorder symptoms in headache patients Headache 2005 14 1365 1374 10.1111/j.1526-4610.2005.00269.x 16324169

[B39] Peterlin BL Tietjen G Meng S Lidicker J Bigal M Posttraumatic stress disorder in episodic and chronic migraine Headache 2008 14 517 522 10.1111/j.1526-4610.2008.00917.x 18377377

[B40] Guidetti V Galli F Fabrizi P Giannantoni AS Napoli L Bruni O Trillo S Headache and psychiatric comorbidity: clinical aspects and outcome in an 8-year follow-up study Cephalalgia 1998 14 455 462 10.1046/j.1468-2982.1998.1807455.x 9793697

[B41] Lantéri-Minet M Radat F Chautard MH Lucas C Anxiety and depression associated with migraine: influence on migraine subjects’ disability and quality of life, and acute migraine management Pain 2005 14 319 326 10.1016/j.pain.2005.09.010 16289799

[B42] Pompili M Serafini G Innamorati M Serra G Dominici G Fortes-Lindau J Pastina M Telesforo L Lester D Girardi P Tatarelli R Martelletti P Patient outcome in migraine prophylaxis: the role of psychopharmacological agents Patient Relat Outcome Meas 2010 14 107 118 22915957 10.2147/PROM.S9742 PMC3417910

